# Recombinant human collagen-based microspheres mitigate cardiac conduction slowing induced by adipose tissue-derived stromal cells

**DOI:** 10.1371/journal.pone.0183481

**Published:** 2017-08-24

**Authors:** Nicoline W. Smit, Judith N. ten Sande, Mojtaba Parvizi, Shirley C. M. van Amersfoorth, Josée A. Plantinga, Carolien A. F. M. van Spreuwel-Goossens, Elisabeth M. W. M. van Dongen, Pascal F. H. M. van Dessel, Sebastianus G. J. M. Kluijtmans, Veronique M. F. Meijborg, Jacques M. T. de Bakker, Martin C. Harmsen, Ruben Coronel

**Affiliations:** 1 Heart Center, Department of Clinical and Experimental Cardiology, Academic Medical Center Amsterdam, University of Amsterdam, Amsterdam, Netherlands; 2 Netherlands Heart Institute, Holland Heart House, Utrecht, Netherlands; 3 Department of Pathology and Medical Biology, University Medical Center Groningen, University of Groningen, Groningen, Netherlands; 4 Fujifilm Manufacturing Europe BV, Tilburg, Netherlands; 5 L’institut de rythmologie et de modélisation cardiaque (LIRYC), Fondation Université Bordeaux, Bordeaux, France; SPAIN

## Abstract

**Background:**

Stem cell therapy to improve cardiac function after myocardial infarction is hampered by poor cell retention, while it may also increase the risk of arrhythmias by providing an arrhythmogenic substrate. We previously showed that porcine adipose tissue-derived-stromal cells (pASC) induce conduction slowing through paracrine actions, whereas rat ASC (rASC) and human ASC (hASC) induce conduction slowing by direct coupling. We postulate that biomaterial microspheres mitigate the conduction slowing influence of pASC by interacting with paracrine signaling.

**Aim:**

To investigate the modulation of ASC-loaded recombinant human collagen-based microspheres, on the electrophysiological behavior of neonatal rat ventricular myocytes (NRVM).

**Method:**

Unipolar extracellular electrograms, derived from microelectrode arrays (8x8 electrodes) containing NRVM, co-cultured with ASC or ASC loaded microspheres, were used to determine conduction velocity (CV) and conduction heterogeneity. Conditioned medium (Cme) of (co)cultures was used to assess paracrine mechanisms.

**Results:**

Microspheres did not affect CV in control (NRVM) monolayers. In co-cultures of NRVM and rASC, hASC or pASC, CV was lower than in controls (14.4±1.0, 13.0±0.6 and 9.0± 1.0 vs. 19.5±0.5 cm/s respectively, p<0.001). Microspheres loaded with either rASC or hASC still induced conduction slowing compared to controls (13.5±0.4 and 12.6±0.5 cm/s respectively, p<0.001). However, pASC loaded microspheres increased CV of NRVM compared to pASC and NRMV co-cultures (16.3±1.3 cm/s, p< 0.001) and did not differ from controls (p = NS). Cme of pASC reduced CV in control monolayers of NRVM (10.3±1.1 cm/s, p<0.001), similar to Cme derived from pASC-loaded microspheres (11.1±1.7 cm/s, p = 1.0). The presence of microspheres in monolayers of NRVM abolished the CV slowing influence of Cme pASC (15.9±1.0 cm/s, p = NS vs. control).

**Conclusion:**

The application of recombinant human collagen-based microspheres mitigates indirect paracrine conduction slowing through interference with a secondary autocrine myocardial factor.

## Introduction

Stem cell-based therapy is an experimental clinical therapeutic option to improve cardiac function and remodeling in post-myocardial infarction patients[1–4]. Poor retention and survival of administered cells[5, 6] are known to limit the therapeutic efficiency of cell-based therapy. Secondly, potential pro-arrhythmic effects of administrated cells or e.g. their secreted molecules are of concern but are inadequately investigated[7–10]

Compared to adult cardiomyocytes that have a resting membrane potential of about -90 mV, mesenchymal stem cells are relatively depolarized, with a resting membrane potential of approximately -35 mV[11, 12]. Therefore, direct—electrotonic- coupling between stem cells and cardiac myocytes will exert electrophysiological effects that alter the electrophysiology that can result in conduction slowing [7, 8, 10] and reentrant arrhythmias [13, 14].

The rationale to employ mesenchymal stem cells in cardiac therapy is their potent paracrine capacity[15]. There is consensus that the hemodynamic benefit of stem cells on cardiac function seen in several pre-clinical post-(acute) myocardial infarction studies is mediated by paracrine signaling. However, little is known about the paracrine interaction [16–18] between stem cells and cardiomyocytes and the possible effects on the electrophysiology[7, 10].

We have recently shown that adipose tissue-derived stromal cells (ASC) cause heterogeneous conduction slowing in cultured neonatal rat ventricular myocytes (NRVM) monolayers. Interestingly, we observed species-dependent differences. Rat ASC (rASC) and human ASC (hASC) altered conduction velocity (CV) via direct—electrotonic- interaction, while porcine ASC (pASC) slowed conduction of NRVM in a paracrine fashion[10]. Conditioned medium of pASC alone induced conduction slowing, suggesting secreted factors from pASC induced the observed conduction slowing [10].

**Fig 1:**
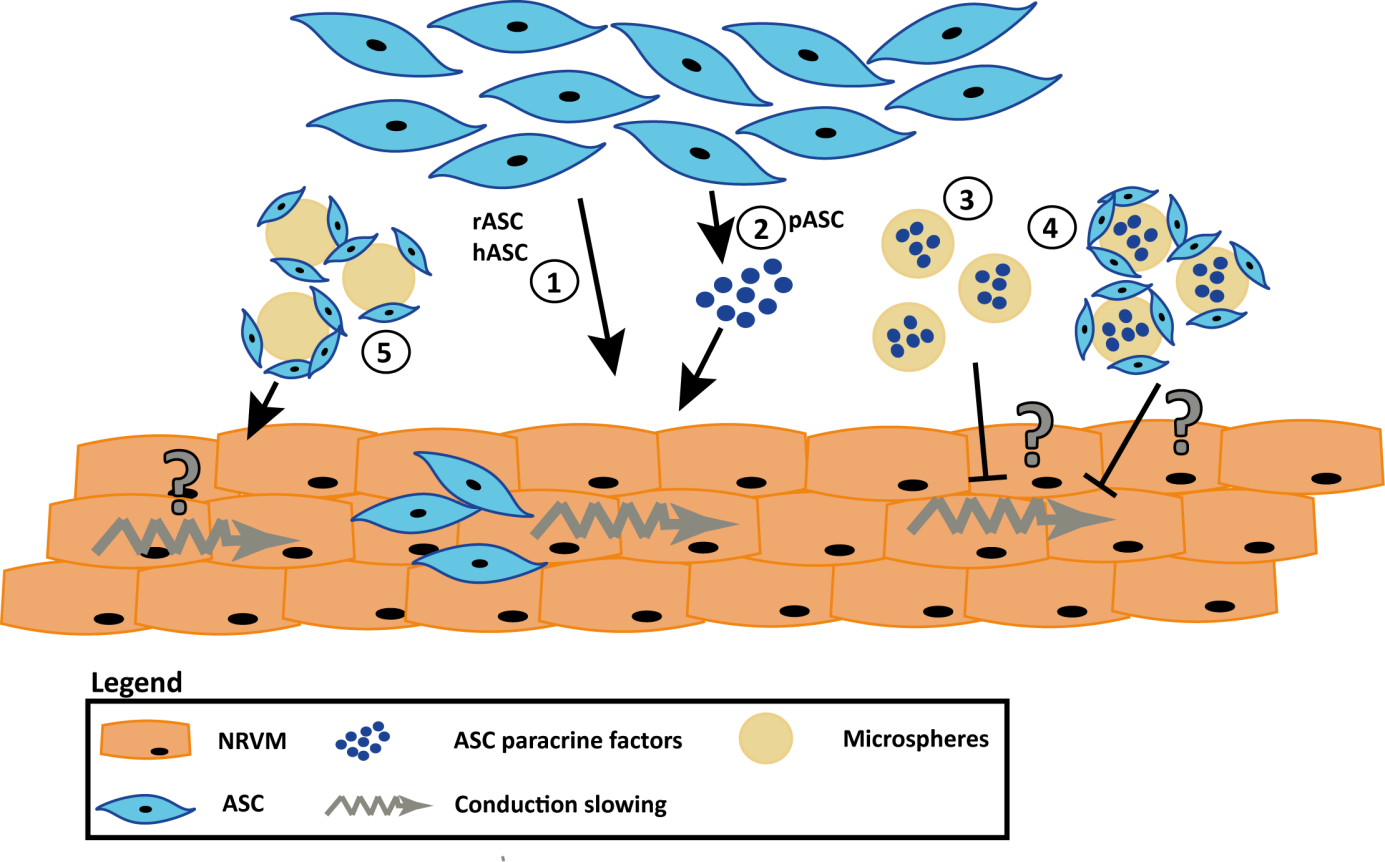
Schematic overview of the hypothesis that recombinant human collagen-based microspheres mitigate the conduction slowing induced by pASC. From previous work we know that rASC and hASC induce conduction slowing through direct-electrotonic-coupling (**1**) and that pASC paracrine factors alone can induce conduction slowing in NRVM monolayers (**2**). We hypothesize that the microspheres will buffer the pASC paracrine factors, and mitigate the conduction slowing (**3**). The mitigating effect can also be achieved when the pASC are loaded onto the microspheres (**4**). When rASC or hASC are loaded onto the microspheres conduction slowing will still occur as these cells induce conduction slowing through direct-electrotonic-coupling(**5**). Abbreviations: hASC: human adipose tissue-derived stromal cells, NRVM; neonatal rat ventricular myocytes, pASC: porcine adipose tissue-derived stromal cells, and rASC: rat adipose tissue-derived stromal cells.

The delivery of stem cells loaded onto or captured inside biomaterials is widely explored to improve cell retention, engraftment as well as function (recently reviewed in[19]). Presently, biomaterials such as hydrogels[20], scaffolds[21], patches[22] and microspheres[23, 24] are used to investigate improvements in retention and engraftment. Because specific microspheres, based on collagen, have been shown to absorb factors (e.g. bone morphogenetic protein-2 and basic fibroblast growth factor) and release this over time[25, 26], we hypothesized that the myocardial conduction slowing effects caused by paracrine interaction between myocytes and ASC derived from porcine are mitigated in the presence of these microspheres. The mitigated effects are expected to be absent in co-cultures of NRVM and ASC derived from rat and man, as these cells induced conduction slowing through direct–electrotonic-coupling (Fig 1).

Similar to mesenchymal stem cells, cardiomyocytes have complex paracrine and autocrine signaling networks[27]. It is therefore plausible that these autocrine networks are affected by pASC-secreted factors, resulting in changes that can influence e.g. conduction velocity. For that reason, by selective exposure of cardiomyocytes to the paracrine factors in the conditioned media, of pASC, in the presence or absence of our microspheres we aimed to identify the source of the paracrine factor responsible for conduction slowing, being either the ASC (primary, direct paracrine effect) and/or the myocytes (secondary indirect autocrine effect).

We used recombinant human collagen-based microspheres (MS), similar to those used in our earlier studies that showed an excellent time-dependent degradation and remodeling *in vivo*[23]. Furthermore these MS can be loaded with ASC, without changing their biological properties such as proliferation rate and secreted factors[24]. The current study demonstrates that specific biomaterial guided stem cell application mitigates pro-arrhythmic paracrine conduction slowing through interference with a yet unidentified, secondary autocrine myocardial factor.

## Materials and methods

All animal experiments were approved by the local Animal Experiments Committee (Academic Medical Center, University of Amsterdam (permit number DCA-192AA1) and carried out in accordance with national and institutional guidelines. Human ASC were obtained from liposuction material (Bergman Clinics, The Netherlands), and was considered as anonymized waste material. However, every anonymized donor gave his/her informed consent for scientific research. Porcine subcutaneous abdominal fat (male, 3–4 months) was provided by the Department of Experimental Surgery of the Academic Medical Center. Inguinal rat fat (male, Wistar, 7–8 months) was provided by the Department of Pathology and Medical Biology, University Medical Center Groningen. In both cases the animals were used in different experiments and this tissue was considered waste material.

### Preparation of neonatal rat ventricular myocyte monolayers

Neonatal rat ventricular myocytes (NRVM) were isolated and cultured as described previously[10]. Briefly, hearts were obtained from one to two days old Wistar rats. Atria were removed and the ventricles, once cut into pieces, were dissociated with trypsin (1 mg/mL; Becton Dickinson BV, Breda, The Netherlands) and collagenase type 2 (1 mg/mL, Worthington Vollenhove, The Netherlands, 230 units/mg). Fibroblast contamination was minimized by a two hour pre-plating step. Ventricular myocytes were then seeded at 1.4 x 10^5^/cm^2^ density onto fibronectin (125 μg/ml BD Biosciences, Breda, The Netherlands) coated microelectrode arrays (MEAs; Multi Channel Systems MCS GmbH, Reutlingen, Germany). This array has 60 integrated extracellular electrodes aligned in an 8 by 8 matrix at interelectrode distances of 0.7 mm (S1 Fig). NRVM were cultured in culture medium (M199 medium; Gibco) supplemented with 10% heat inactivated fetal bovine serum (FBS; Gibco), 1% HEPES (Gibco #5630-0-80), 5000 U/L penicillin-G (Sigma,#P7794), 2 mg/L vitamin B12 (Sigma, #V2876), 3.5 g/L glucose, 1% non-essential amino acids (Gibco, #11140–050), and 1% L-glutamine (Gibco,#25030–081). Light microscopy was used to determine if a confluent (and spontaneous beating) monolayer had formed in each of the cultures. A total of 24 cell isolations were performed to execute the experiments.

### Isolation and culture of adipose tissue-derived stromal cells

Porcine, rat and human adipose tissue derived stromal cells (ASC) were isolated and cultured as previously described[10, 28, 29]. Briefly, collected tissue was minced and washed in phosphate buffered saline before the dissociation steps with collagenase A (0.1%, Roche Diagnostics, Mannheim, Germany). The obtained stromal vascular fraction was incubated with erythrocyte lysis buffer before the cells were seeded at a density of 4 x 10^4^ cells/cm^2^. Culture medium consisted of DMEM (Lonza Biowhittaker, Verviers, Belgium), supplemented with 10% FBS (Thermo Scientific, Hemel Hempstead, UK), 100 U/mL penicillin, 100 mg/mL streptomycin (Gibco, Invitrogen, Carlsbad, CA) and 2 mM L-glutamine (Lonza Biowhittaker,Verviers, Belgium). ASC were propagated at a 1:2 ratio and used from passage 3 onwards.

### Experimental conditions

Mitomycin-C (Sigma M4287-2MG) treated ASC were labeled with CDFA-SE (Invitrogen Vybrand® CFDA SE Cell Tracer Kit) before they were collected with accutase. ASC were added to four days old monolayers of NRVM in cell ratio of NRVM:ASC 1:1. On the same day monolayers of NRVM serving as controls received fresh culture medium containing 2% FBS. Two days later electrical mapping was performed. The co-cultures were named NRVM + ASC indicating that NRVM and ASC were cultured together.

### Preparation of ASC-loaded recombinant human collagen-based microspheres

Recombinant human collagen-based microspheres (MS) were provided by Fujifilm[23, 24]. Microspheres were prepared from an RGD enriched human collagen I derived recombinant peptide (commercially available under the tradename Cellnest™(Fuji Film). Details of microsphere production, size and structure can be found in the S1 Appendix.

Of the MS, 10 mg was incubated in 10 mL ASC culture medium and stored at 4°C until MS were loaded with ASC. Mitomycin-C-treated and SDFA-SE (Invitrogen Vybrand® CFDA-SE Cell Tracer Kit)-labeled ASC were loaded onto MS (at 2.5 x 10^5^ ASC per mg) in low-adherent T6 wells with ASC culture medium and left overnight at 37°C in 5% CO_2_. The following day the cell/microsphere suspension was collected and centrifuged at 160 g for 5 minutes. Supernatant was removed and the pellet was re-suspended in an equal amount of NRVM culture medium containing 2% FBS, the cell/microsphere suspension was then added to the appropriate monolayers; the NRVM:ASC ratio used was 1:1.

Monolayers of NRVM with bare MS (NRVM/ MS) were made on the same day as co-cultures and two days later electrical measurements were performed. The different co-cultures were named NRVM + ASC/MS indicating that NRVM and ASC loaded microspheres were cultured together.

### Conditioned medium

Different types of conditioned media were used. Conditioned medium (Cme) was removed, after 48 hours, from confluent cultures of pASC or cultures consisting of pASC loaded MS, filtered (0,22 μm) and stored at -20°C until use. This medium was referred to as Cme [pASC] and Cme [pASC/MS] respectively. Cme was also removed, after 48 hours, from monolayers of NRVM containing pASC loaded MS, this medium was filtered (0.22μm) and stored at -20°C until use, and was referred to as Cme [NRVM + pASC/MS].

Monolayers of NRVM/ MS were incubated with Cme [pASC] and received this medium 8 hours after microspheres were added to give the microspheres an opportunity to sediment on the NRVM monolayer.

### Electrical mapping and microelectrode measurements

Electrical mapping was performed as previously described[10]. Briefly, cultures grown on multi-electrode arrays were stimulated, on day 6 of culture, using a bipolar extracellular stimulus electrode (twice diastolic stimulation threshold, 1 or 2 ms rectangular current pulses).

At each local electrogram activation time (AT) was determined as the interval from the stimulation artefact to the minimum derivative of the local QRS. The minimum derivative (dV/dt) threshold was set at -0.1 mV/ms. From these AT activation maps were constructed. Conduction velocity (CV) was determined along lines perpendicular to isochronal lines by dividing the distance by the difference in local activation time. Lines had a length of at least 4 electrode distances[10]. Based on the method described by Lammers et al.[13] we quantified the heterogeneity in conduction. Resting membrane potential was taken as the highest negative membrane potential recorded and Vmax (upstroke velocity) was taken as dV/dt max. All signal analysis were performed using a custom-made data analysis program written in Matlab 2006b (The MathWorks Inc, Natick, MA)[30]. For each experiment (cell isolation) two monolayers of NRVM only served as control. Monolayers that were not beating spontaneously or un-excitable when stimulated were discarded.

Following activation mapping, action potentials were recorded in control cultures, co-cultures of NRVM and pASC loaded microspheres and monolayers of NRVM cultured in Cme pASC. Microelectrodes were pulled from borosilicate glass capillaries (Harvard apparatus GC100F-10) and filled with 3 M KCl. An AgCl covered silver wire was used as a reference electrode. Typical tip resistance was 15 and 25 MΩ.

### Immunostainings

Cells plated in 12-wells plate used for immunofluorescence were cultured under the same conditions and timing as cells on MEAs. Cells were permeabilized after fixation in 4% PFA and blocked before staining with primary antibodies (rabbit anti Connexin 43 (Invitrogen 574366A; 1:200) and mouse anti N-Cadherin (Sigma C2542, 1:100)) and secondary antibodies (Alexa Fluor-488 goat anti-mouse IgG (Life Technology, A21235; 1:250), Alexa Fluor-647 goat anti- rabbit IgG (Life Technology, A11008/A21222; 1:250)). Nuclei were stained with DAPI (Sigma, D9542, 1:40000). Examination was performed by Leica SPE confocal laser scanning and Leica Application Suite Advanced Fluorescence software. Immunofluorescence images were analyzed using Image J version 1.50i (National Institutes of Health, NIH, USA) as described previously[10].

### Statistical analysis

Data that were continuous and normally distributed are presented as mean ± SEM, and compared using an independent *t*-test. In case of more than two groups an one-way ANOVA was performed with the Bonferroni correction as post-hoc analysis. The conduction heterogeneity data showed a skewed distribution and hence are presented as median with the interquartile range (IQR) and tested with the Mann Whitney U test. In case of more than two groups a Kruskal-Wallis analysis was performed with post-hoc analysis using the Dunn’s test. A p-value of <0.05 was considered significant.

## Results

### Cell characterization

**Fig 2:**
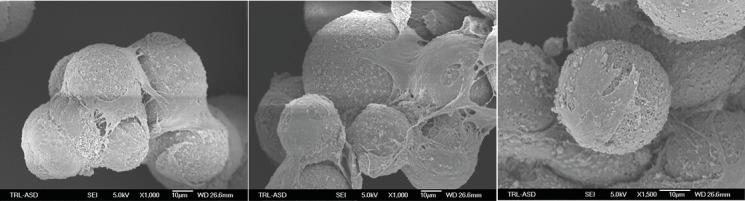
Scanning electron microscope micrographs of the recombinant human collagen-based microspheres loaded with porcine ASC. Original magnification is indicated underneath the image. Abbreviation: ASC: adipose tissue-derived stromal cells.

**Fig 3:**
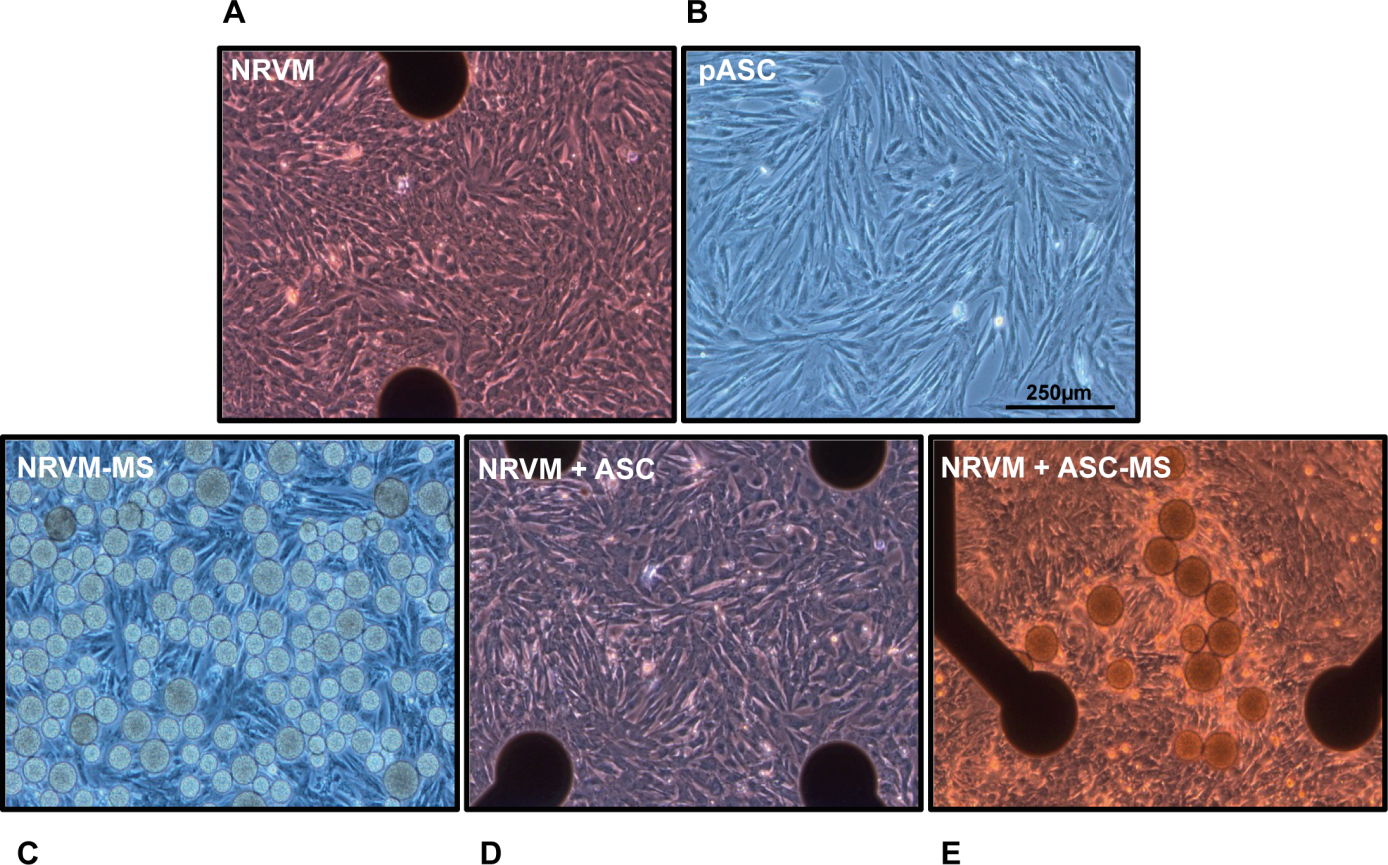
Light microscope image of the two main cell types used and the different cultures. **A:** Monolayers of NRVM cultured on multi-electrode arrays, **B:** Porcine ASC in culture, **C:** Monolayers of NRVM cultured together with microspheres, **D:** Co-cultures of NRVM and porcine ASC on a multi-electrode array and **E:** Co-cultures of NRVM and porcine ASC loaded on microspheres. Original magnification 10x. Black dots represent electrode terminals in the multi-electrode arrays. Abbreviations; ASC: adipose tissue-derived stromal cells, MS: microspheres and NRVM: neonatal rat ventricular myocytes.

Scanning electron microscopy of ASC-loaded MS, revealed that ASC had adhered and spread over the surface (Fig 2) of the recombinant human collagen-based MS. Adhered ASC also bridged multiple microspheres. No signs of apoptosis (e.g. blebbing) were observed. The micrographs obtained with the scanning electron microscope also show the rough surface and porous nature of the microspheres. Confluent monolayers of NRVM cultured with and without ASC and ASC-loaded MS were visualized with light microscopy at day six (Fig 3).

### Influence of recombinant human collagen-based microspheres on conduction parameters in monolayers of NRVM

**Fig 4:**
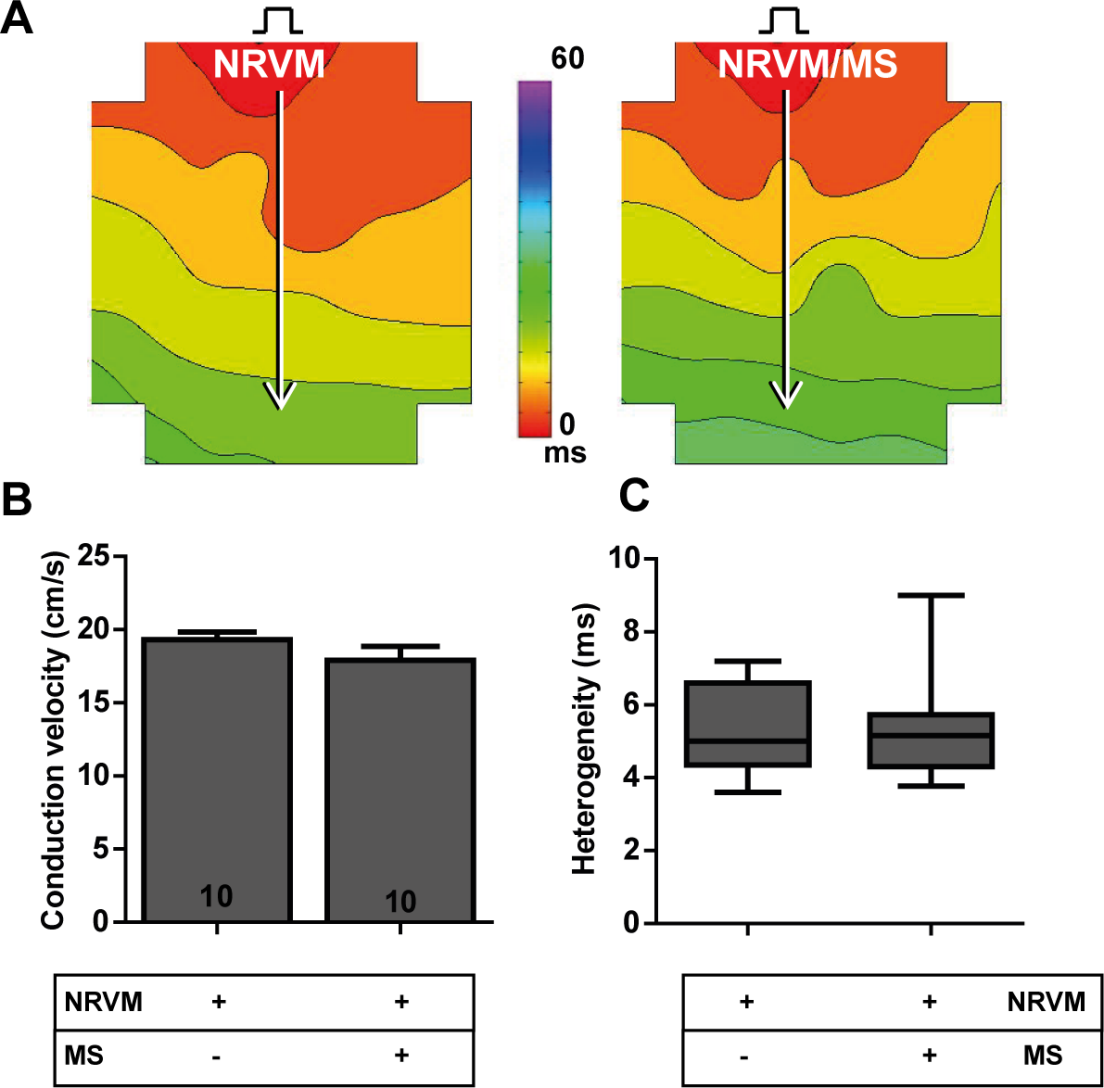
Effects of recombinant human collagen-based microspheres on NRVM monolayers. **A:** An activation map of a monolayer of NRVM (left) and a monolayer of NRVM cultured with microspheres (right). Conduction velocities are determined along the white arrows perpendicular to isochronal (black) lines. The effects of culturing bare microspheres on monolayers of NRVM on **B:** conduction velocity and **C:** conduction heterogeneity. Number of replicates per experimental condition is indicated in the bar-graphs. Abbreviations; MS: microspheres and NRVM: neonatal rat ventricular myocytes.

Monolayers of NRVM showed normal, homogeneous propagation (Fig 4A). Monolayers of NRVM cultured with bare microspheres also demonstrated normal, homogeneous propagation. The CV in monolayers of NRVM in contact with bare microspheres (NRVM/MS) did not differ from control monolayers without microspheres (17.9±0.9 vs. 19.3±0.5 cm/s, NRVM/MS and NRVM respectively, Fig 4B). Conduction heterogeneity in monolayers of NRVM cultured with MS also did not differ from controls (5.2 (1.4) vs. 5.0 (2.3) ms, NRVM/MS and NRVM respectively, Fig 4C).

### Influence of recombinant human collagen-based microspheres on conduction slowing mediated by a paracrine mechanism

**Fig 5:**
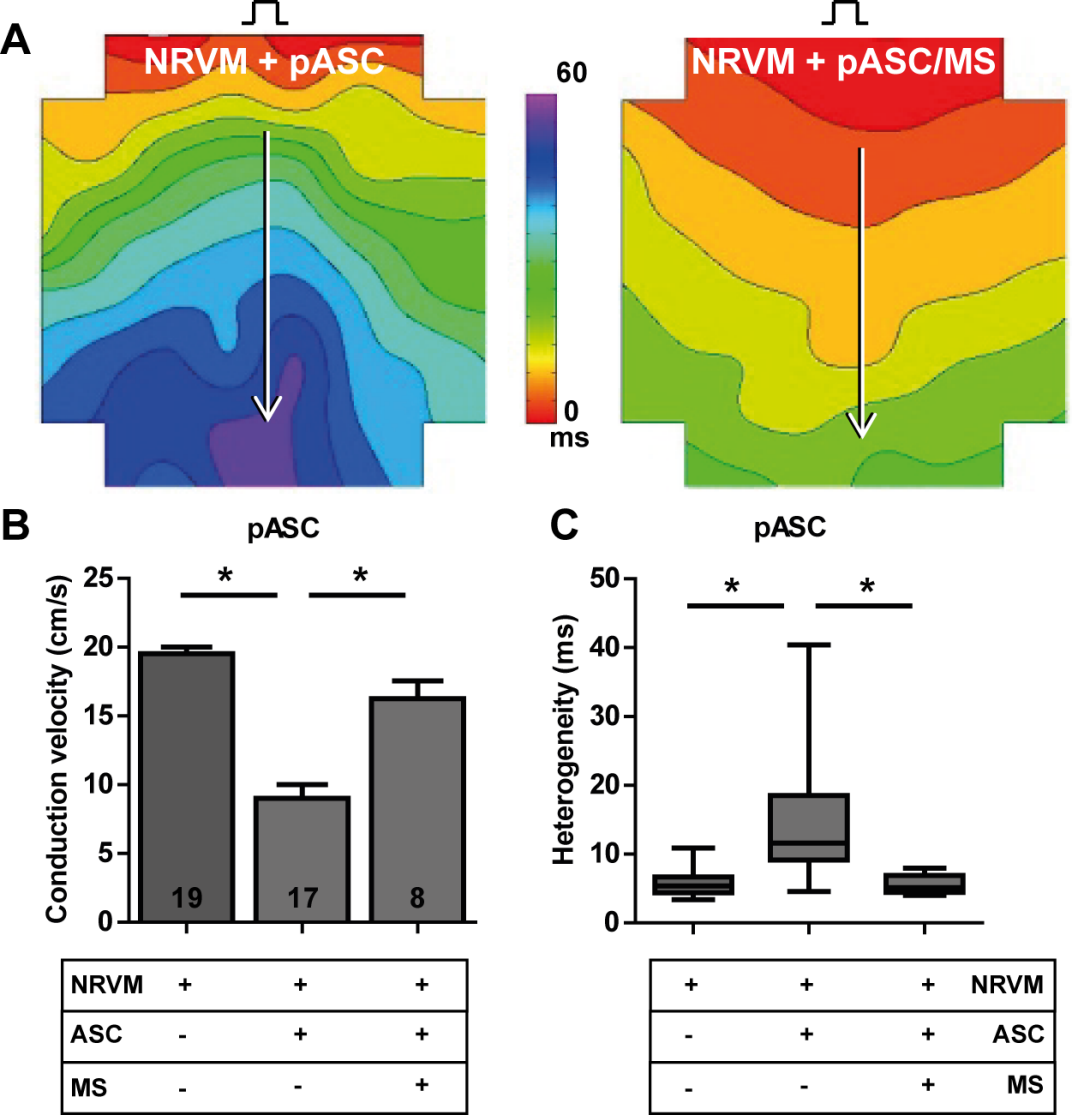
Influence of recombinant human collagen-based microspheres on conduction slowing mediated by a paracrine mechanism. **A:** Activation map of a monolayer of NRVM co-cultured with pASC (left), and an activation map of a monolayer of NRVM cultured with pASC loaded microspheres (right). Conduction velocity is determined along the white arrows perpendicular to isochronal (black) lines. Lower panels: the effects on **B:** conduction velocity and **C:** conduction heterogeneity. * indicates p< 0.001 compared to the NRVM + pASC. Number of replicates per experimental condition is indicated in the bar-graphs. Abbreviations; MS: microspheres, NRVM: neonatal rat ventricular myocytes, and pASC: porcine adipose tissue-derived stromal cells.

Co-cultures of NRVM and pASC showed heterogeneous conduction slowing compared to the control monolayers studied on the same day. Conduction velocity was 9.0±1.0 cm/s in co-cultures (NRVM + pASC) compared to 19.5±0.5 cm/s in control monolayers (NRVM, p<0.001, Fig 5B). Conduction heterogeneity was increased in the co-cultures with pASC relative to controls (11.6 (9.4) vs. 5.4 (2.3) ms, NRVM + pASC and NRVM respectively, p<0.001, Fig 5C).

In monolayers co-cultured with pASC loaded MS (NRVM+pASC/MS) conduction was similar to control (NRVM) monolayers. Overall, conduction velocity in NRVM+pASC/MS was 16.3±1.3 cm/s vs. 19.5±0.5 cm/s, in control cultures (NRVM, p = 0.08, Fig 5B). Conduction heterogeneity in NRVM+pASC/MS was 5.1 (2.5) vs. 5.4 (2.3) ms in control cultures (NRVM, p = 0.92, Fig 5C). Both conduction velocity and heterogeneity differed significantly from co-cultures with bare pASC (NRVM + pASC, CV: 9.0±1.0 cm/s, p < 0.001, heterogeneity: 11.6 (9.4) ms, p<0.01, Fig 5B and 5C).

**Fig 6:**
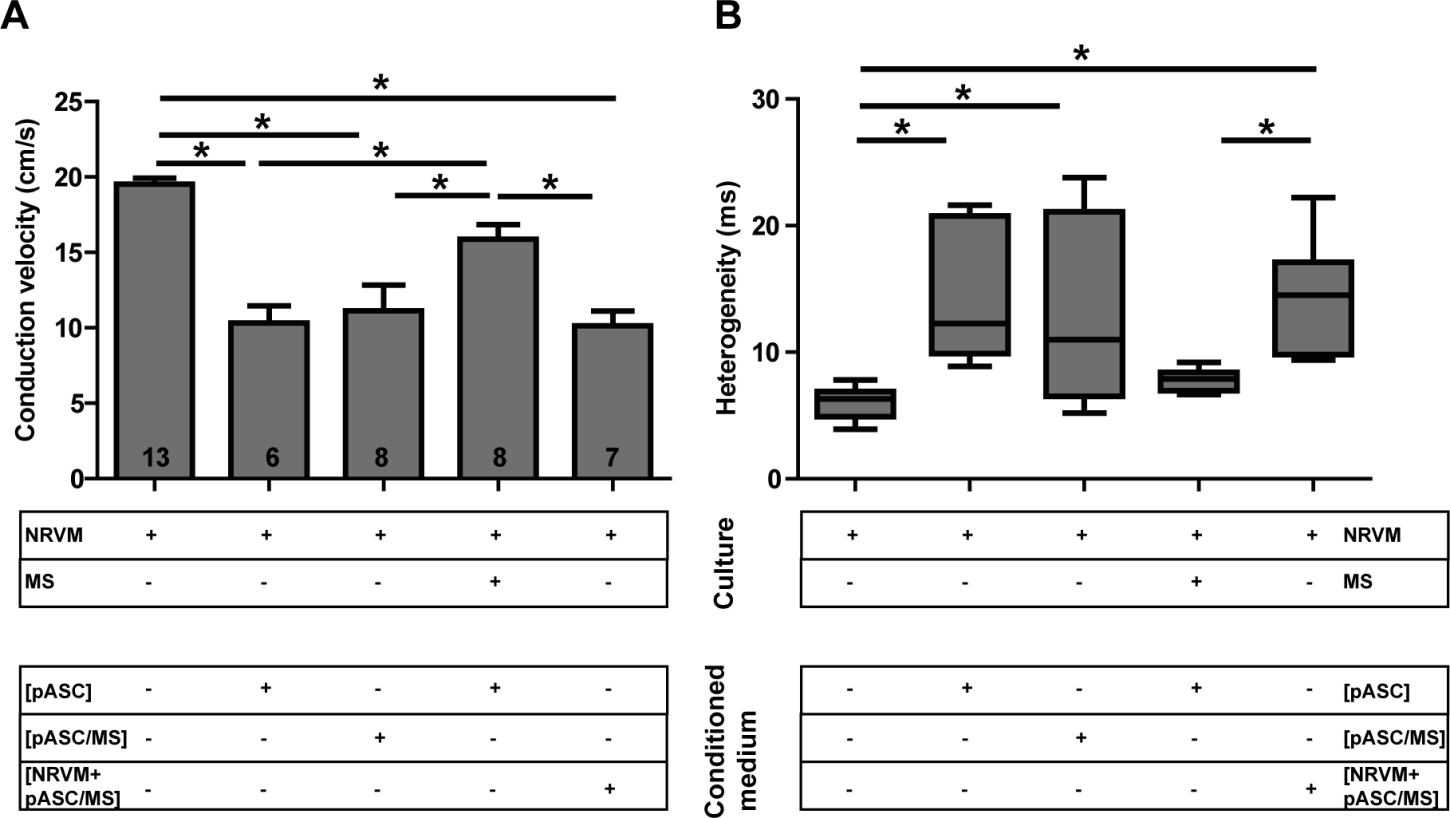
Effects of conditioned medium. The effects on **A:** conduction velocity and **B:** conduction heterogeneity of monolayers of NRVM cultured in various conditioned mediums. * indicates p≤ 0.05. Number of replicates per experimental condition is indicated in the bar-graphs. Abbreviations; Cme: conditioned medium, MS: microspheres, NRVM: neonatal rat ventricular myocytes and pASC: porcine adipose tissue-derived stromal cells. See text for details.

Conditioned medium obtained from pASC cultures induced conduction slowing and increased conduction heterogeneity (Fig 6, 2^nd^ bar), as demonstrated before[10]. We tested whether the mitigated effect of the microspheres (Fig 5) was related to primary paracrine effects derived from the porcine stem cells (pASC). To test this, conditioned medium obtained from cultures of pASC- loaded microspheres was applied to control NRVM monolayers. In these monolayers conduction velocity was also lower than in controls (11.1±1.7 vs. 19.5±0.4 cm/s, p< 0.001, Fig 6A, 3^rd^ bar) and conduction heterogeneity was higher than in controls (10.9 (14.6) vs. 6.3 (2.0) ms, p<0.01, Fig 6B). Conduction velocity and its heterogeneity did not differ between monolayers of NRVM cultured in conditioned medium of pASC or those cultured in conditioned medium of pASC loaded microspheres (Fig 6, 2^nd^ and 3^rd^ bar).

The experiments so far showed that the conduction slowing effect of pASC in monolayers of NRVM is based on a paracrine effect and that this effect (conduction slowing) is absent in the presence of the microspheres in co-cultures of NRVM and pASC (Fig 5B, 3^rd^ bar). However, conduction velocity is not normalized in monolayers of NRVM cultured in conditioned medium obtained from cultures of pASC-loaded microspheres (Fig 6, 3^rd^ bar). This suggested that the microspheres did not buffer the paracrine mediators of the pASC as we originally assumed. Thus, we hypothesized that conduction slowing is generated from the cardiomyocytes as a secondary or indirect paracrine effect induced by the pASC paracrine factors and that the microspheres interfere with this secondary myocardial factor. It also implies that the microspheres should be in direct contact with the NRVM in order to suppress the conduction slowing effect of the ASC conditioned medium.

We tested this by incubating monolayers of NRVM with microspheres prior to exposing these to conditioned medium obtained from pASC. In these cultures conduction velocity did not differ from control NRVM monolayers (15.9±1.0 vs. 19.5±0.4, p = 0.1, Fig 6A, 4^th^ bar). Similarly, heterogeneity did not differ between these two conditions (7.8 (1.45) vs. 6.3 (2.0), p > 0.1, Fig 6B). Conduction velocity under these conditions, however, differed significantly from monolayers of NRVM cultured in conditioned medium of pASC and conditioned medium of pASC-loaded microspheres (compare to Fig 6A, 2^nd^ and 3^rd^ bar). Thus, the paracrine mediators responsible for conduction slowing are derived from NRVM secondary to exposure to Cme pASC. We surmise that the recombinant human collagen-based microspheres neutralize these NRVM-derived paracrine mediators.

We further tested this by deriving conditioned medium from co-cultures of NRVM and pASC loaded microspheres and adding this conditioned medium to a control culture of NRVM. We expected that the primary, porcine derived paracrine factors contained in the Cme would maintain its effect on the NRVM monolayer, leading to a secondary autocrine effect resulting in conduction slowing. Fig 6 (last bar) shows that under these conditions, conduction was slower than control and did not differ from cultures of NRVM cultured in conditioned medium of pASC (Fig 6A, last and 2^nd^ bar). Conduction heterogeneity demonstrated the corresponding effects (Fig 6B).

#### Upstroke velocity of action potentials and Cx43

**Fig 7:**
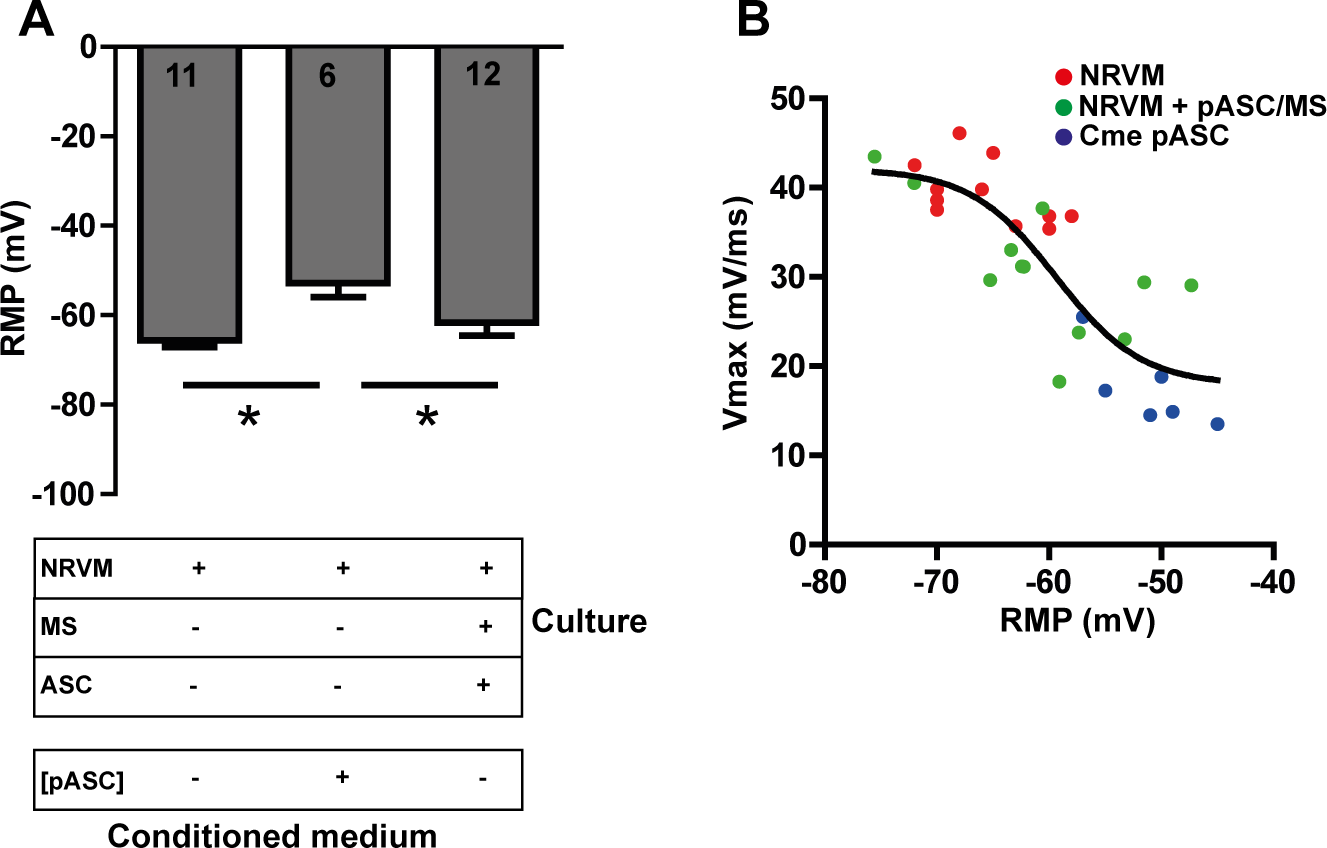
The relationship between RMP and Vmax. **A:** The effect of conditioned medium and pASC loaded microspheres on the RMP. **B:** The relationship between RMP and Vmax in the different cultures * indicates p≤ 0.05. Number of cells analyzed is indicated in the bar-graphs. Abbreviations; Cme: conditioned medium, MS: microspheres, NRVM: neonatal rat ventricular myocytes, pASC: porcine adipose tissue-derived stromal cells, and RMP: resting membrane potential.

The degree of depolarization is a main determinant in conduction velocity and a theoretical sigmoid relationship between RMP and Vmax (upstroke velocity) exists[31, 32]. From the previous study we observed that ASC depolarized the co-cultures[10]. Here, we recorded action potentials from monolayers of NRVM co-cultured with pASC loaded microspheres, monolayers of NRVM cultured in conditioned medium and control monolayers. Conditioned medium of pASC depolarized monolayers (-51.2±1.8mV) compared to control monolayers (-65.6±1.5, p<0.01, Fig 7A) and monolayers cultured with pASC loaded microspheres (-61.6±2.9 mV, p<0.01). The resting membrane potential did not differ between control monolayers and monolayers cultured with pASC loaded microspheres (p = ns, Fig 7A). We next looked at the relation between RMP and Vmax in these three cultures. Fig 7B shows a sigmoid function fitted through the combined data points (black line). Because the average data do not appear to deviate from the theoretical sigmoid function the figure show that the degree of depolarization is the main determinant of the Vmax and that the relation was not different between the groups as was seen before[10].

**Fig 8:**
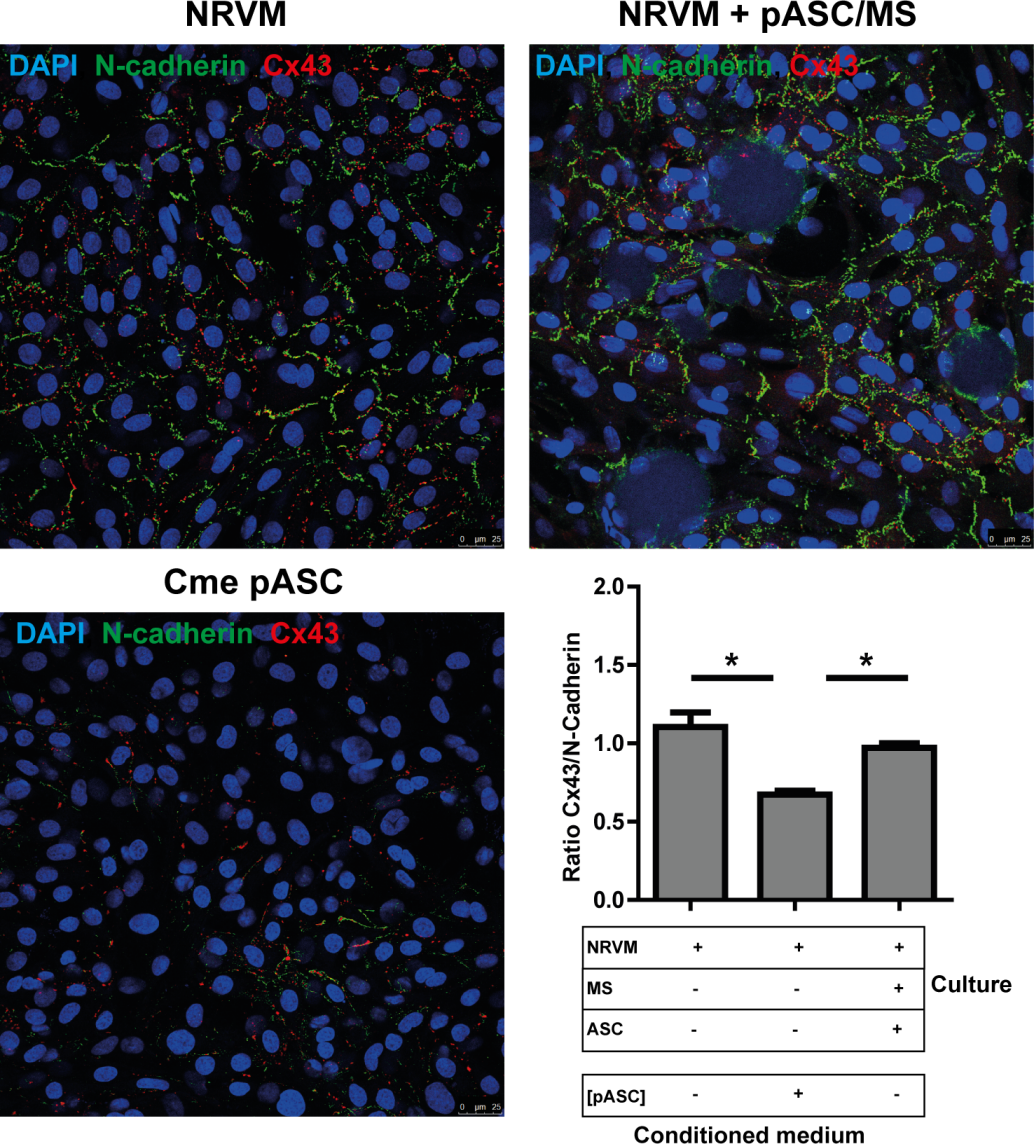
Immunofluorescence micrographs of cultures stained with N-cadherin and Cx43. **A:** A monolayer of NRVM, a monolayer of NRVM cultured in pASC conditioned medium and a monolayer of NRVM co-cultured with pASC loaded microspheres are stained for N-Cadherin and Cx43. **B:**The ratio of Cx43: N-Cadherin in the various cultures, determined by the number of pixels. Ratios are based on 5–10 images taken in each of three independent cultures. * indicates p< 0.05. Abbreviations; Cme: conditioned medium, Cx43: connexin 43, MS: microspheres, NRVM: neonatal rat ventricular myocytes and pASC: porcine adipose tissue-derived stromal cells.

We previously demonstrated that the ratio of Cx43/N-Cadherin was lower in co-cultures of NRVM and pASC[10]. In the present study we used control cultures and co-cultures of NRVM and pASC loaded microspheres and NRVM cultured in pASC conditioned medium to determine the ratio of Cx43/N-Cadherin. The ratio of Cx43/N-Cadherin in the co-culture with pASC loaded microspheres (0.97±0.03) did not differ from control monolayers (1.10±0.10, p = ns, Fig 8). Monolayers of NRVM cultured in pASC conditioned medium demonstrated significantly lower Cx43/N-Cadherin ratio (0.67±0.03) compared to controls (p<0.001) and co-cultures with pASC loaded microspheres (p<0.001).

### Influence of recombinant human collagen-based microspheres on conduction slowing mediated by contact based mechanism

**Fig 9:**
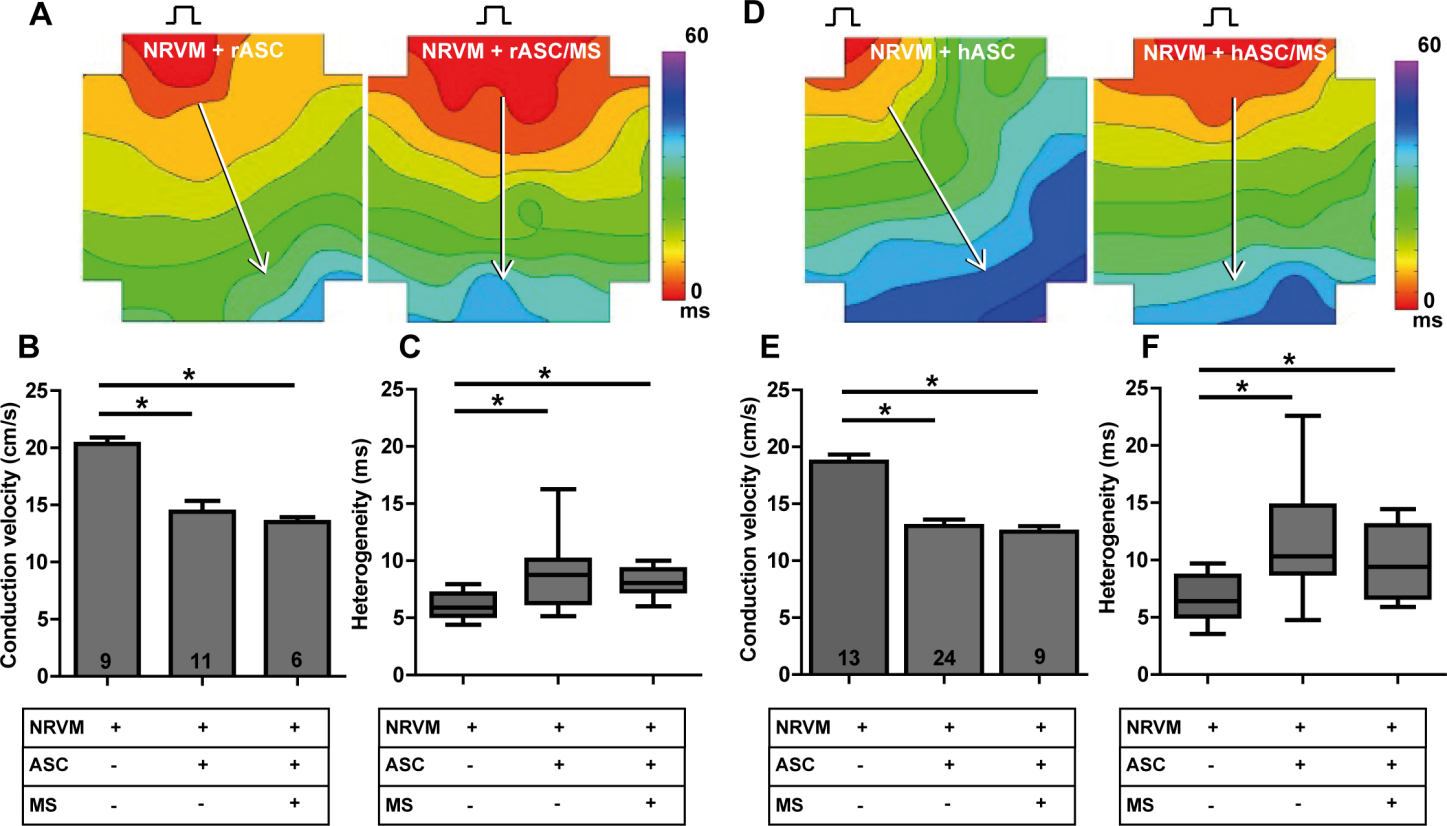
Influence of recombinant human collagen-based microspheres on conduction slowing mediated by a contact based mechanism. Activation maps of **A:** monolayers of NRVM cultured with rASC and rASC loaded microspheres, respectively. The effect on conduction velocity (**B**) and conduction heterogeneity (**C**) are determined along the white arrows perpendicular to isochronal (black) lines. Activation maps of **D**: monolayers of NRVM cultured with hASC and hASC loaded microspheres, respectively. The effect on conduction velocity (**E**) and conduction heterogeneity (**F**) are determined along the white arrows perpendicular to isochronal (black) lines.* indicates p< 0.001 compared to the monolayers of NRVM. Number of replicates per experimental condition is indicated in the bar-graphs. Abbreviations; hASC: human adipose tissue-derived stromal cells, MS: microspheres, NRVM: neonatal rat ventricular myocytes, and rASC: rat adipose tissue-derived stromal cells.

**Fig 10:**
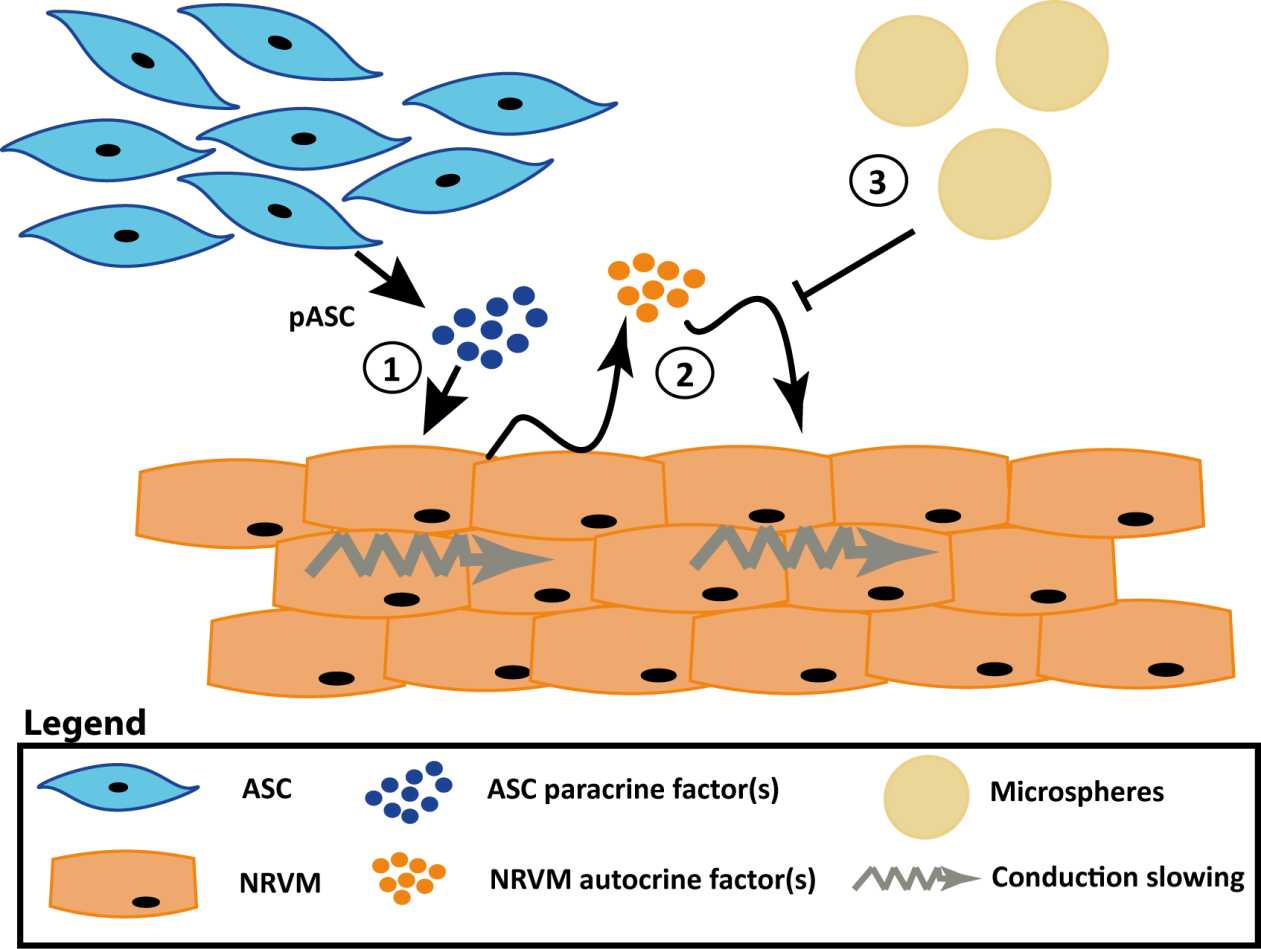
Schematic illustration of how the recombinant human collagen-based microspheres mitigate the conduction slowing induced indirectly by porcine adipose tissue-derived stromal cells. (**1**) pASC secrete (a) paracrine factor(s) that induce NRVM to produce (a) secondary autocrine factor(s) that is responsible for the observed heterogeneous conduction slowing (**2**). When the microspheres are added to monolayers of NRVM, either bare or loaded with pASC, the secondary autocrine myocardial factor is buffered by the microspheres and the conduction slowing is mitigated (**3**). Abbreviations; NRVM: neonatal rat ventricular myocytes and pASC: porcine adipose tissue-derived stromal cells.

Rat ASC. Monolayers of NRVM co-cultured with rat ASC (rASC) showed decreased conduction velocity and increased heterogeneity compared to control monolayers (Fig 9B, [10]). When rASC-loaded microspheres were used, conduction velocity and heterogeneity did not differ from co-cultures containing bare rASC (13.5±0.4 vs.14.4±1.0 cm/s and 8.1 (1.9) vs. 8.8 (3.8) ms,Fig 9B and 9C, 3^rd^ bar vs. 2^nd^ bar). However, compared to control monolayers, conduction velocity was decreased (13.5±0.4 vs. 20.3±0.6 cm/s, p<0.001, respectively, Fig 9B, 1^st^ bar vs. 3^rd^ bar) and heterogeneity had increased (8.1 (1.9) vs. 5.9 (1.9) ms, p < 0.01, Fig 7C).

Human ASC. Human ASC(hASC) in co-culture with NRVM did not differ from rASC with respect to electrophysiological effects. Monolayers of NRVM co-cultured with hASC showed heterogeneous conduction slowing compared to control monolayers (CV: 13.0±0.6 vs. 18.7±0.6 cm/s respectively, p < 0.001 and heterogeneity: 11.9 (5.9) vs. 6.4 (3.5) ms, p< 0.01, Fig 9E + F, 2^nd^ bar vs. 1^st^ bar) as shown previously[10]. After application of hASC-loaded microspheres heterogeneous conduction slowing was still observed and CV and heterogeneity did not differ from co-cultures containing bare hASC. Compared to control monolayers of NRVM, co-cultures with hASC-loaded microspheres had decreased conduction velocity (12.6±0.5 vs. 18.7±0.6 cm/s, p<0.001, Fig 9E, 3^rd^ bar vs. 1^st^ bar) and increased heterogeneity (9.7 (6.3) vs. 6.4 (3.5) ms, p<0.01, Fig 9F, 3^rd^ vs. 1^st^ bar).

## Discussion

In this study, we investigated whether human recombinant collagen-based microspheres loaded with ASC mitigate the conduction slowing effects of unaccompanied ASC on a confluent layer of neonatal rat ventricular cardiomyocytes. We demonstrated earlier that ASC from three species cause heterogeneous conduction slowing. Human and rat ASC exert a direct-electrotonic-coupling effect, those from porcine exert paracrine effects[10]. In the current study, we show that application of bare microspheres has no effect on conduction properties of NRVM monolayers (Fig 4). This is an important necessity of any biomaterial if it is to be used for clinical myocardial therapy. Secondly, we demonstrated that microspheres loaded with rASC or hASC do not influence the conduction properties of cultured cardiomyocytes differently compared to bare rASC or hASC (Fig 9). In contrast, pASC-loaded microspheres normalized conduction properties of NRVM compared to co-cultures with unaccompanied pASC (Fig 5). Finally, we demonstrated that the mere presence of MS in monolayers of NRVM, abolished the conduction slowing induced indirectly by paracrine pASC factors. We speculate that the MS buffer the secondary autocrine myocardial factor(s) produced by NRVM in response to the pASC secreted factors, schematically summarized in Fig 10.

First, we reasoned that the microspheres used in our experiments acted as an absorbent/adsorbent for the unidentified paracrine mediator(s) released specifically by pASC[10]. It could be argued that the pASC were inactivated by the presence of the microspheres. However, previous work demonstrated that ASC maintain their secretion profile when loaded onto the microspheres[24]. Secondly, when we applied conditioned medium produced by pASC in the presence of microspheres, onto a monolayer of NRVM, heterogeneous conduction slowing was still observed (Fig 6). This indicates that pASC are not inactivated by the microspheres. However, it also implied that the microspheres do not buffer the secreted pASC factors, as we first speculated. We next hypothesized, that the MS buffered the secondary autocrine myocardial factor(s) produced by NRVM in response to the pASC secreted factors. This is supported by the experiments.

Next, we observed that the conduction slowing effect is mitigated when pASC loaded microspheres are cultured together with NRVM (Fig 5). However, when we applied conditioned medium from these co-cultures (Cme [NRVM + pASC/MS]) onto a monolayer of NRVM, heterogeneous conduction slowing was observed (Fig 6). The observations can be explained by hypothesizing the presence of a secondary paracrine factor derived from NRVM. In the first observation, pASC release factors that induce the NRVM to secrete a secondary autocrine myocardial factor(s) which is buffered by the MS. In the second experiment the conditioned medium applied to a new monolayer of NRVM, contains no secondary autocrine myocardial factors, as these were buffered, but does contain pASC paracrine mediators. These pASC derived mediators are now able to induce the NRVM to produce the secondary autocrine myocardial factor again, resulting in conduction slowing, as there were no microspheres to buffer the newly produced secondary factor. This is further strengthened by the experiments in which conditioned medium of pASC was applied to monolayers of NRVM cultured in the presence of the human recombinant collagen-based MS. In these cultures (NRVM/MS + Cme [pASC]), conduction was normalized (Fig 6), as in the presence of the MS the secondary autocrine myocardial factor was buffered.

It is plausible that the mitigating effect of conduction slowing by the recombinant human collagen-based microspheres is caused by preventing physical contact between ASC and the cardiomyocytes. However, we demonstrated before[10] and in the current study, that physical contact between porcine ASC and cardiomyocytes is not required for heterogeneous conduction slowing. When bare pASC or factors secreted from pASC are cultured with NRVM heterogeneous conduction slowing is observed in monolayers of NRVM. Secondly, the scanning electron microscope showed that ASC formed an interconnected layer on and between microspheres indicating that the ASC were connected, most likely by cell adhesion molecules or otherwise adhesive molecules. The binding of ASC to microspheres does not preclude their juxtacrine interaction with NRVM monolayers. Earlier studies from our group, have shown that mesenchymal-like cells in fact do interact via juxtacrine connections with neonatal cardiomyocytes[33].

Data from the microelectrode recordings demonstrated that the relation (typically a sigmoid relation[31, 32]) between RMP and Vmax (upstroke velocity) of the action potentials recorded was not different between the groups. The immune-histochemical stainings (Fig 8) demonstrated that conduction slowing is independent of the presence of (p)ASC(a structural factor), as the ratio of Cx43/N-Cadherin was not changed in the co-culture with pASC loaded microspheres but was lower in the Cme pASC condition. This strongly suggests that functional changes underlie the observed conduction slowing.

Finally, we do not see a mitigating effect on conduction slowing when rASC or hASC are loaded onto the microspheres. This suggests that the previously observed electrotonic influence of rASC and hASC on NRVM is maintained when loaded onto the microspheres.

Taken together the current investigation demonstrates that the factors released by pASC initiate NRVM to secrete secondary–autocrine- factors that cause conduction slowing. Conduction slowing is an important contributor to reentry based arrhythmias[34]. In the presence of our recombinant human collagen-based microspheres, irrespective of loading with pASC, these myocardial autocrine factors are inactivated, absorbed, adsorbed, or buffered.

Whether the beneficial hemodynamic effects of stem cell therapy outweigh the pro-arrhythmic effects remains a topic of debate. It may be argued that the application of a biomaterial leads to a diminution of not only the adverse electrophysiological effects but also of the beneficial effects of stem cells. Our study does not allow conclusions on this aspect. However, a recent paper by de Jong et al., has demonstrated that intracoronary administration of encapsulated mesenchymal stem cells into a myocardial infarct swine model did not diminish the hemodynamic effect of the mesenchymal stem cells[35]. This is important to know because human ASC are now recognized to have a prominent paracrine role in modulating post myocardial infarction inflammation and having augmented hemodynamic effects via paracrine mechanisms[15, 36].

Cardiomyocytes communicate in multiple ways. Direct communications can occur through juxtacrine connections but also via gap junctions. Communication also happens via signaling interactions of the extracellular matrix. Thirdly, paracrine communication, although involving a complex network of soluble factors, plays an important role[27, 37]. Pedrotty et al., investigate paracrine effects of fibroblast on conduction properties of cardiomyocytes. Unidentified factors released by cardiac fibroblasts were able to induce conduction slowing by altering ion channel expression and inducing hypertrophy[38]. Similar results were obtained by Askar et al., who demonstrated that paracrine factors released by mesenchymal stem cells induced conduction slowing and increased re-entry inducibility. Askar et al., further demonstrated that these effects are mediated by directly secreted factors, because mesenchymal stem cell derived exosomes (micro vesicle-associated factors) did not have an effect[7]. In another study on the effects of fibroblast on cardiomyocytes, cardiomyocyte hypertrophy was observed. In that study it was demonstrated that transforming growth factor beta (TGFβ) had an bidirectional regulatory signaling role between the fibroblasts and cardiomyocytes[39]. Factors such as TGFβ are involved or expressed in response to hypertrophy and loss of adhesions. Modifications due to paracrine signaling in e.g. cell size, (gap) junctions and ion channel expression can all result in alterations in conduction.

Our study does not allow identification of the factors released by the pASC nor the NRVM, that indirectly and directly result in conduction slowing, because this would require an extensive proteomics-based approach. However, our data strongly points into the direction of a myocyte derived soluble factor for the generation of conduction slowing. We also emphasize the potential role of biomaterial microspheres as a tool to mitigate adverse electrophysiological effects of ASC.

## Conclusions

Our previous results indicated that ASC cause heterogeneous conduction slowing when cultured on a monolayer of NRVM[10]. Paracrine modulation and intercellular coupling between the ASC and NRVM contribute to the formation of a potentially pro-arrhythmic substrate. Here, we show that application of recombinant human collagen-based microspheres loaded with pASC normalizes the electrophysiological properties of a monolayer of NRVM compared to monolayers of NRVM co-cultured with unaccompanied pASC, through interference/adsorption of an unidentified, secondary, autocrine, myocardial factor. The mitigating effect of these recombinant human collagen-based microspheres is absent in co-cultures with rat and human ASC, that display predominant direct coupling effects.

### Perspectives

The fact that our recombinant human collagen-based microspheres do not influence conduction velocity in monolayers of NRVM is a positive result which does not obstruct cardiac clinical applications of stem cells. We have further shown that there is inherent risk in the use of mesenchymal stem cells for cardiac cell-based therapies and that the mechanisms are species dependent. These clues obtained from *in vitro* work are important for translation towards the clinic and can be used to improve therapy such that the pro-arrhythmic risks are reduced. Although, pASC influence conduction via indirect paracrine effects, hASC do this via direct–electrotonic-interactions. Our data imply that biomaterial guided stem cell therapy may mitigate the potentially electrophysiological pro-arrhythmic side effects of ASC and that it has a possible role in the treatment of cardiovascular disease. In order to e.g. prevent the direct-electrotonic effects of hASC, these cells could be embedded in biomaterial, allowing continuously release of paracrine factors, while not negatively affecting the cardiac electrophysiology.

Therefore, further development of biomaterials as carriers for stem cells for cardiac therapy is warranted. Not only does the ‘behavior’ of stem cells, such as ASC, depend on the nature and 3D-formulation of a biomaterial, but it is plausible to reason that different biomaterials, either cell-loaded or bare, will influence cardiac function at the electrophysiological level, too. In the current study, we showed that the mere presence of recombinant human collagen-based microspheres attenuated the adverse indirect paracrine effects of pASC on conduction properties of cultured cardiomyocytes.

## Supporting information

S1 Fig Layout of the 60 electrodes in the MEA. Each electrode has a diameter of 100 μm and an interelectrode distance of 700 μm. Numbers 1 to 4 represent stimulation positions. Abbreviation: MEA: multi-electrode array.
(TIF)

S1 Appendix Supplementary data. Specific details on microsphere production and electrical mapping.
(DOC)
