# Supplementary figures and images for: Recombinant human collagen-based microspheres mitigate cardiac conduction slowing induced by adipose tissue-derived stromal cells
Source: PLoS One. 2017 Aug 24;12(8):e0183481. doi: 10.1371/journal.pone.0183481 (PMC5570323; doi:10.1371/journal.pone.0183481)

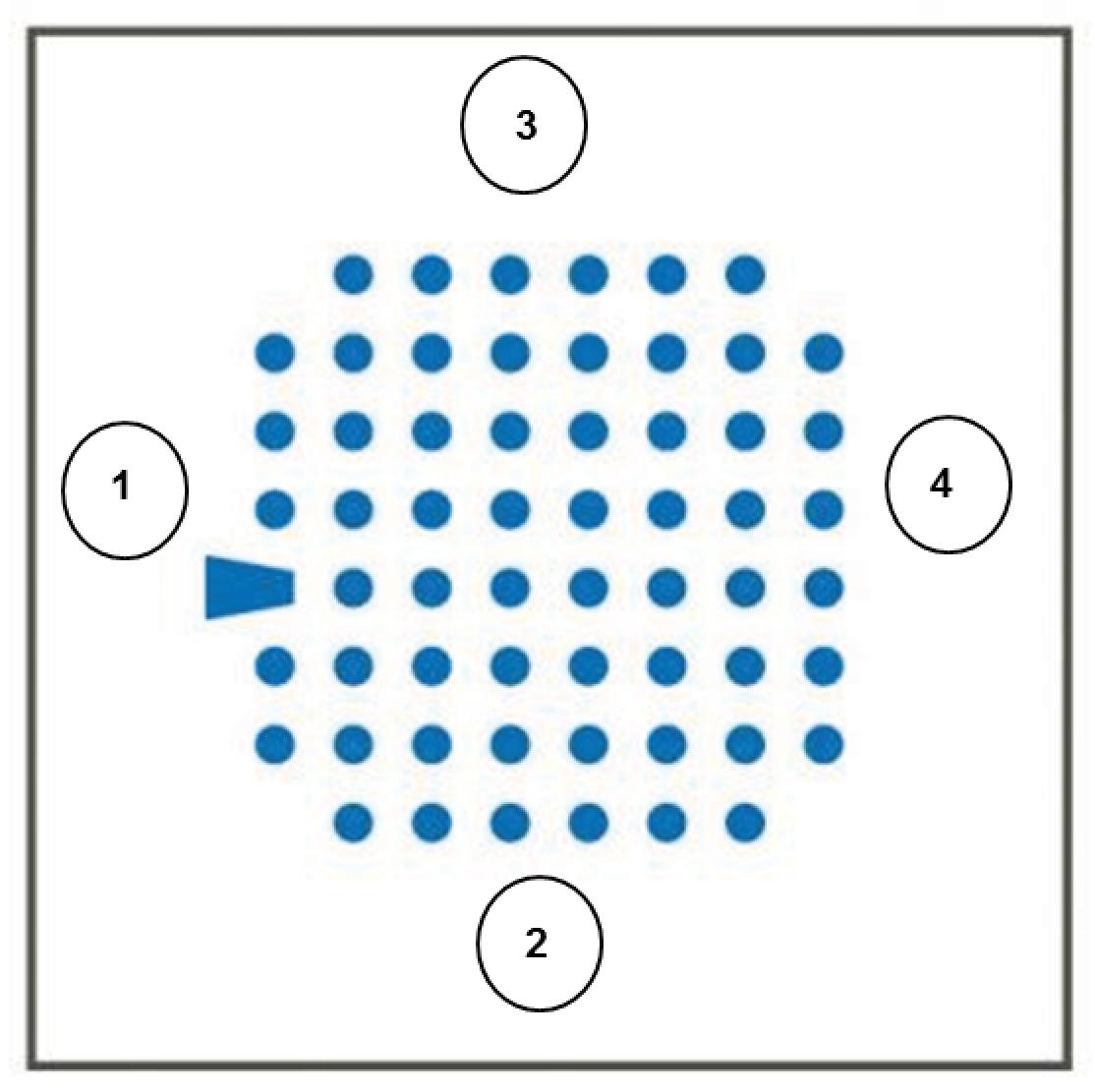

Supplement: S1 Fig — Each electrode has a diameter of 100 μm and an interelectrode distance of 700 μm. Numbers 1 to 4 represent stimulation positions. Abbreviation: MEA: multi-electrode array. (TIF) [file pone.0183481.s001.tif]
