# Supplementary material for: Recombinant human collagen-based microspheres mitigate cardiac conduction slowing induced by adipose tissue-derived stromal cells
Source: PLoS One. 2017 Aug 24;12(8):e0183481. doi: 10.1371/journal.pone.0183481 (PMC5570323; doi:10.1371/journal.pone.0183481)
Supplement: S1 Appendix — Specific details on microsphere production and electrical mapping. (DOC) [file pone.0183481.s002.doc]

**S1 Appendix. [Supplementary data].** [Specific details on microsphere production and electrical mapping]

Methods

***Microsphere production, loading, size and structure***

Microspheres based on a human collagen type I derived recombinant peptide (MS) were provided by Fujifilm. The RCP having a sequence of 243 amino acids. Rough MS were prepared via water in oil emulsification using calcium carbonate as a porogen. Briefly, calcium carbonate with a crystal size of 1 µm (Acros) was mixed in a 1:1 ratio with 20-w/w% recombinant collagenous peptide solution. This mixture was subsequently emulsified in corn oil by thoroughly mixing for 20 minutes. MS were harvested by cooling to 4 °C and were rinsed with acetone for several times to remove the oil. Finally, the MS were dried overnight at 60 °C. Microspheres were subsequently cross-linked by high concentrations of hexamethylene diisocyanate (HMDIC). Calcium carbonate was removed by acid washing steps with 1 M HCl. MS were again washed with buffer and water to remove residual crosslinking agents and dried overnight at 60 °C. MS were sieved to yield microspheres sized between 50 and 100 µm. A scanning electron microscope was used to visualize MS loaded with ASC.

***Electrical mapping*** MEAs containing the different cultures were positioned in a temperature controlled (37^o^C) MEA holder (TC01/02 Multichannel Systems MSC GmbH). Each MEA had an organization of 60 electrodes which had terminals in the core portion of the MEA (Supplemental Fig.1). All cultures were stimulated from at least two stimulation sites using a bipolar extracellular stimulus electrode (twice diastolic stimulation threshold, 1 ms or 2 ms rectangular current pulses). Unipolar electrograms were recorded with a 256-channel amplifier (BioSemi, ActiveTwo, Amsterdam, The Netherlands, 24 bit dynamic range, 122.07 nV LSB, total noise 0.5 µV). Signals were recorded with a sampling frequency of 2048 Hz (filter setting of the amplifiers DC – 400 Hz (- 3dB point). Conduction velocity (CV) was determined from activation maps constructed using the maximum negative dV/dt as activation time (AT; relative to the time of earliest activation) with the use of a custom made program[1] based on MATLAB R2006b (The MathWorks, Inc., Natick, MA, USA). CV was determined along lines perpendicular to isochronal lines by dividing the distance by the difference in local activation time. Lines had a length of at least 4 electrode distances. Local activation times in the figures are color coded in classes of 5 ms. CV was determined at a basic cycle length (BCL) of 600 ms or during spontaneous activity if pacing was not possible. Based on the method described by Lammers et al.[2] we quantified the heterogeneity in conduction as a measure of arrhythmia vulnerability. Maximum AT differences between each adjacent electrode quartet in the grid were obtained and the total range of maximal AT differences was plotted in a histogram.

References

[1] Potse M, Linnenbank AC, Grimbergen CA. Software design for analysis of multichannel intracardial and body surface electrocardiograms. Comput Methods Programs Biomed 2002;69:225-36.

[2] Lammers WJ, Schalij MJ, Kirchhof CJ, Allessie MA. Quantification of spatial inhomogeneity in conduction and initiation of reentrant atrial arrhythmias. Am J Physiol 1990;259:H1254-63.
